# Antibacterial and Antifungal Properties of Composite Polyethylene Materials Reinforced with Neem and Turmeric

**DOI:** 10.3390/antibiotics9120857

**Published:** 2020-11-30

**Authors:** Thefye P. M. Sunthar, Elia Marin, Francesco Boschetto, Matteo Zanocco, Hirofumi Sunahara, Raviduth Ramful, Kaeko Kamei, Wenliang Zhu, Giuseppe Pezzotti

**Affiliations:** 1 Ceramic Physics Laboratory, Kyoto Institute of Technology, Sakyo-ku, Matsugasaki, Kyoto 606-8585, Japan; d0871502@edu.kit.ac.jp (T.P.M.S.); boschetto-cesc@kit.ac.jp (F.B.); d8871004@edu.kit.ac.jp (M.Z.); wlzhu@kit.ac.jp (W.Z.); pezzotti@kit.ac.jp (G.P.); 2 Department of Immunology, Graduate School of Medical Science, Kyoto Prefectural University of Medicine Kamigyo-ku, 465 Kajii-cho, Kawaramachi Dori, Kyoto 602-0841, Japan; 3 Department of Dental Medicine, Graduate School of Medical Science, Kyoto Prefectural University of Medicine, Kamigyo-ku, Kyoto 602-8566, Japan; 4 Department of Biomolecular Engineering, Kyoto Institute of Technology, Sakyo-ku, Matsugasaki, Kyoto 606-8585, Japan; m9674018@edu.kit.ac.jp (H.S.); kame@kit.ac.jp (K.K.); 5 Graduate School of Science and Technology, Kyoto Institute of Technology (KIT), Matsugasaki, Sakyo-ku, Kyoto 606-8585, Japan; r.ramful@uom.ac.mu; 6 Mechanical and Production Engineering Department, Faculty of Engineering, University of Mauritius, Reduit 80837, Mauritius; 7 The Center for Advanced Medical Engineering and Informatics, Osaka University, Yamadaoka, Suita, Osaka 565-0871, Japan; 8 Department of Orthopedic Surgery, Tokyo Medical University, Tokyo 105-8461, Japan

**Keywords:** turmeric, neem, antibacterial, antifungal, polyethylene

## Abstract

With the increased scientific interest in green technologies, many researches have been focused on the production of polymeric composites containing naturally occurring reinforcing particles. Apart from increasing mechanical properties, these additions can have a wide range of interesting effects, such as increasing the resistance to bacterial and fungal colonization. In this work, different amounts of two different natural products, namely neem and turmeric, were added to polyethylene to act as a natural antibacterial and antifungal product for food packaging applications. Microscopic and spectroscopic characterization showed that fractions of up to 5% of these products could be dispersed into low-molecular weight polyethylene, while higher amounts could not be properly dispersed and resulted in an inhomogeneous, fragile composite. In vitro testing conducted with *Escherichia coli*, *Staphylococcus aureus*, and *Candida albicans* showed a reduced proliferation of pathogens when compared to the polyethylene references. In particular, turmeric resulted in being more effective against *E. coli* when compared to neem, while they had similar performances against *S. aureus*. Against *C. albicans*, only neem was able to show a good antifungal behavior, at high concentrations. Tensile testing showed that the addition of reinforcing particles reduced the mechanical properties of polyethylene, and in the case of turmeric, it was further reduced by UV irradiation.

## 1. Introduction

Foodborne illnesses are a serious problem worldwide. Statistics from the Centers for Disease Control and Prevention report that one in six Americans suffer from foodborne illnesses, such as *Salmonella, Norovirus, Listeria, and Escherichia coli* each year [1], resulting in about 3000 deaths [2] and thousands of chronic and debilitating permanent conditions such as kidney failure, paralysis, or rheumatoid arthritis [3].

Annual reports of food poisoning cases around the world can be correlated with various factors such as climate [4], average income [5], and food miles [6]. In higher income countries, food quality controls are usually stricter, and even minor changes in aspect or smell can easily result in withdrawal [7].

A forth key parameter is represented by “food hygiene”, which strongly depends on education. A survey conducted on food handlers in the plantation sector of Sri Lanka in 2016 showed that only about 60% of the 375 interviewed had good knowledge of food practice and hygiene, and these results could be well correlated with the levels of education [8]. A 2018 systematic review on the literature on street food research [9] showed that out of 441 total papers (of which 3/4 were from Africa or Asia), 376 were partially or completely focused on food safety. The review also showed how the amount of research done on this topic has been constantly increasing in the last 20 years. However, similar issues were also observed at supermarkets and stores around Europe and in the United States [10].

Other than education, a major role is also played by the perception of risk: producers often tend to underestimate the levels of risk represented by their products, focusing more on quality [11], while managers running businesses on a limited budget try to reduce time and resources spent on training and food safety [12].

The problem of food hygiene is not limited to businesses: consumers were demonstrated to be optimistically biased when considering the chances of food poisoning from self-prepared foods [13]. General guidelines about food hygiene can be found in the “*Codex Alimentarius*” distributed by the Food and Agriculture Organization of the United Nations [14].

One of the most common sources of food poisoning arises from improperly cleaned and dried equipment [15] such as mincers, slicers, cutlery, food processors, as well as food packaging materials [16].

Polyethylene is the most common synthetic plastic material used in the world, accounting for about 34% of the total plastic market (100 million tonnes, annually). This is due to the simple nature of its basic chemical structure, composed of ethylene groups (C${}_{2}$H${}_{4}$) and a low melting temperature (about 115 ${}^{{}^{\circ}}$C) [17].

Polyethylene has low strength, hardness, and rigidity, but high ductility and impact strength and a low friction coefficient against most materials. Under constant load, it is easily subjected to high creep due to its low melting temperature.

Many grades of polyethylene are commonly used in industry, ranging from low- (LDPE) to medium- (MDPE) or even high-density (HDPE), depending on the structure.

LDPE is defined by a density range of 0.917–0.930 g/cm^3^. It has more branching (about 2% of the carbon atoms) than HDPE, which results in weaker intermolecular forces, lower tensile strength, and higher resilience. Furthermore, because its molecules are less tightly packed and less crystalline due to the side branches, its density is lower. It is mainly applied in containers, corrosion-resistant surfaces, weldable parts, juice and milk cartons (where it is applied as a film), plastic wraps, and toys.

For many years, packaging materials have been chosen solely by using three criteria; compatibility with food, stability at various conditions, and manufacturing costs [18].

Recent trends towards greener technologies have pushed plastic companies and researchers to develop more environmentally friendly products, which can be bio-degraded [19], composted [20], or recycled [21]. Since these solutions have usually lower mechanical properties or higher costs, producers are trying to compensate by adding additional features, such as antibacterial capabilities [22]. Antibacterial (or bacteriostatic) packaging can increase both shelf life and food miles when applied to products. Turmeric is a flowering plant, Curcuma longa of the ginger family, Zingiberaceae, the rhizome of which is used as a cooking spice. The main bioactive molecule in turmeric is curcumin ((1E,6E)-1,7-Bis(4-hydroxy-3-methoxyphenyl)hepta-1,6-diene-3,5-dione), and it has been investigated as a natural antibacterial, antiviral, and antifungal agent [23,24]. Curcumin has two main forms (Figure S1), diketone and keto-enol, which have different colors due to the presence of three (or two) carbon double bonds along the main chain [24].

Neem is the common name for the tree Azadirachta indica, in the mahogany family Meliaceae. Neem is typically grown in tropical and sub-tropical regions where it is used in traditional medicine, as well as in clothes to protect against insect attack. It contains various bioactive chemicals, of which only two were reported to have antibacterial and antifungal properties, namely quercetin [25] and sitosterol [26] (Figure S1).

In this research, turmeric and neem powders were added to polyethylene to produce bioactive composites with antibacterial and antiviral properties. The bioactive effects were tested against Gram positive (*S. aureus*) and Gram negative (*E. coli*) bacteria and *C. albicans* fungi. Mechanical testing was also conducted to evaluate the effects of the particles on the strength of polyethylene and the material reaction to UV light exposure.

## 2. Materials and Methods

### 2.1. Samples Production

Both turmeric and neem powders were obtained by harvesting the respective plants in Malaysia. The raw materials were then mechanically ground until an average particle size of 50 μm was obtained. Polyethylene pellets were obtained from an industrial producer (Sigma-Aldrich, Tokyo, Japan). The two materials were mixed by using a mechanical stirrer for 30 min at a speed of 0.5 Hz. Mixed powders were then put into silicon molds and melted in a vacuum oven at a temperature of 200 ${}^{{}^{\circ}}$C.

The composition of the samples is listed in Table 1.

### 2.2. Samples’ Characterization

#### 2.2.1. Laser Microscopy

The surface morphology of the samples before and after biological testing was analyzed using a confocal scanning laser microscope (Laser Microscope 3D and Profile measurements, Keyence, VK × 200 Series, Osaka, Japan). All images were collected at various magnifications ranging from 10× to 150×. Initial roughness values were measured on 10 randomized 100 × 100 μm square areas per sample.

#### 2.2.2. Fourier Transformed Infrared Spectroscopy

Fourier Transformed Infra-Red spectroscopy (FTIR) spectra were collected at room temperature using an FTIR spectrometer (FT/IR-4000 JASCO, Tokyo, Japan) equipped with a Michelson 28 deg interferometer with corner-cube mirrors, covering a range between 250,000 and 5 cm${}^{-1}$. The aperture size was 200 × 200 μm^2^, and the acquisition time was set to 30 s. The instrument was operated using dedicated software (Spectra Manager, JASCO, Tokyo, Japan). Ten different spectra were acquired for each sample before and after biological testing. The average spectra are presented, unless otherwise specified.

#### 2.2.3. In Vitro Bacteria and Fungi Cultures

*S. aureus* (NBRC 13276), *E. coli* (E1 NBRC 3972), and *C. albicans* (NBRC 1594) were cultured by the streak plate method. The first plate was prepared using bacteria beads in the agar plate. The second plate was prepared through four sector quadrant streaks using one colony from the first plate. Both plates were incubated under 37 ${}^{{}^{\circ}}$C for 24 h. Subsequently, one colony collected from the second plate was taken and mixed with 5 mL of Luria broth (LB Broth, Sigma-Aldrich, Tokyo, Japan) and Broth n° 108 formulated by mixture of glucose, peptone, yeast extract, malt extract, distilled water (NBRC, NITE biological resource center, Shibuya-ku, Tokyo, Japan). LB broth was used for S. aureus and *E. coli*, and 108 broth was used for *C. albicans*, then incubated on a shaking incubator (Southwest Science, Hamilton, NJ, USA) at 37 ${}^{{}^{\circ}}$C and 175 rpm for 24 h. Finally, one-hundred microliters of the bacteria solution were pipetted into new LB broth, and the dilution steps were repeated until the absorbance of 0.3 OD was achieved using the miniphoto 518R photometer (TAITEC CORPORATION, Tokyo, Japan).

### 2.3. Biological Characterization

#### Colony Forming Units

Two-hundred microliters of the bacterial and fungal solutions were dropped onto the surface of the samples (5 cm × 5 cm) and covered with parafilm (4 cm × 4 cm). Each sample was replicated three times to calculate the average. Then, the samples were arranged in a container and incubated for 24 h at 37 ${}^{{}^{\circ}}$C. After the incubation period, the samples were washed with 5 mL of sterilized phosphate-buffered saline (PBS) (Fujifilm WakoCo, Osaka, Japan). Then, one-hundred microliters were taken out from the mixture and placed into the microcentrifuge tube for 10-fold serial dilution from 10${}^{-1}$ to 10${}^{-7}$ by PBS. Finally, one-hundred microliters of each dilution were spread on an agar plate and incubated at 37 C for 24 h, which was followed by counting the number of colonies of the respective dilution. The CFU/mL was calculated using the formula: CFU/mL = (no. of colonies × dilution factor) × 10. Then, the CFU values were multiplied by the polyethylene surface and divided by the area of the parafilm used.

### 2.4. Aging and Mechanical Testing

#### 2.4.1. UV-Aging

The samples with dimensions of 1 cm × 1 cm were treated in UV Form Cure (Formlabs, Somerville, MA, USA) equipped with 13 LEDs at 405 nm. The temperature was maintained at 25 ${}^{{}^{\circ}}$C for 0 h, 15 min, 1 h, 6 h, 24 h, and 1 week. The changes in the sample’s color were observed, and FTIR analysis was performed. Each sample was replicated three times to calculate the average value.

#### 2.4.2. Mechanical Testing

The tensile testing of the samples in dumbbell shapes was performed using the MCT 2150 Desktop Tensile-Compression Tester (AND Discover Precision, Tokyo, Japan) at an elongation rate of 50 mm/min. The tensile properties were determined for three samples of each concentration.

## 3. Results

### Surface Characterization

**Figure 1:**
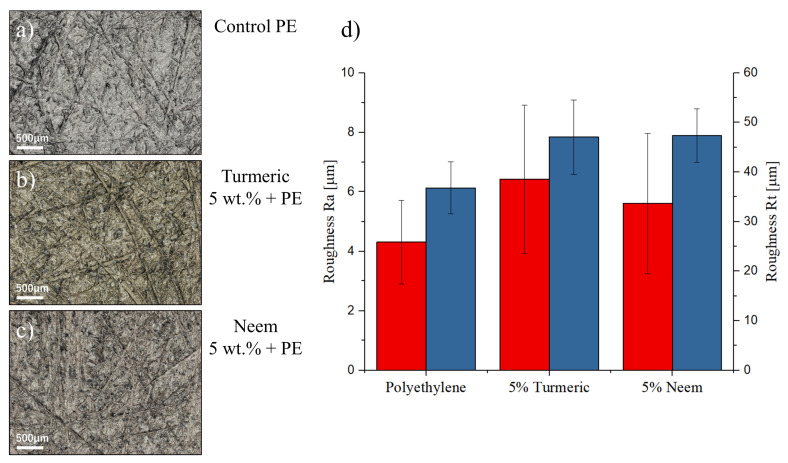
Optical micrographs of (**a**) control PE, (**b**) PE containing 5% of turmeric, and (**c**) PE containing 5% of neem. (**d**) shows the surface roughness of the different samples.

Figure 1 shows the results of the surface characterization as performed with laser microscopy. It can be observed that the three materials have similar scratches, which were caused by the marks on the surface of the silicon molds used to produce them. The presence of reinforcing particles up to 5% (Figure 1b,c) did not affect the surface morphology observable by optical microscopy.

Figure 1d shows the results of the surface roughness measurements performed on the reference polyethylene sample and on the two composites with the highest fraction of reinforcements. It can be observed that even if the average roughness (Ra) values slightly increase, the standard deviation is still large, and the differences between the three groups are not significant (*p* > 0.05). Similarly, the total height of the maximum roughness amplitude (Rt) is increased with the addition of reinforcing particles, but even if the differences are statistically significant between the reference and the 5% neem samples, the increase is relatively small (about 20%).

**Figure 2:**
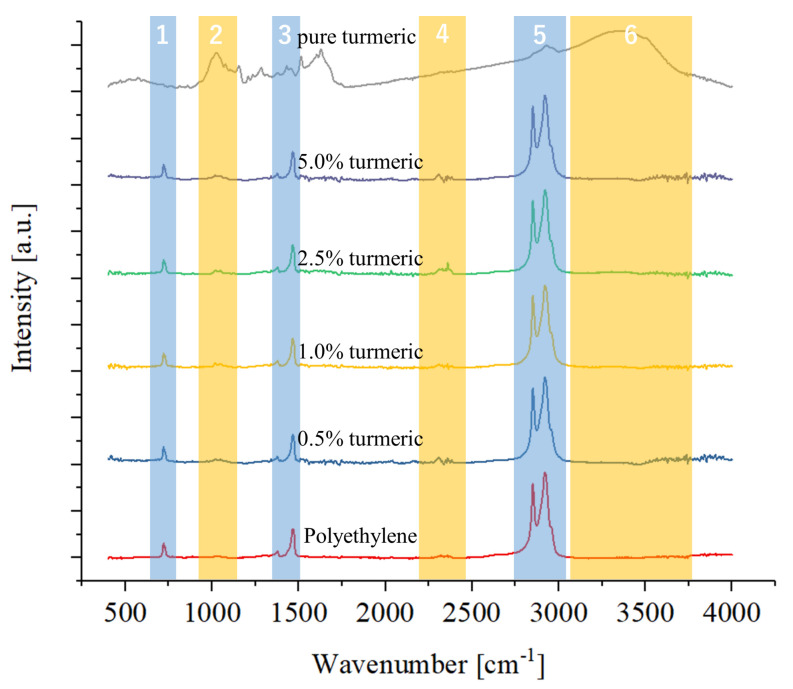
FTIR spectra of the samples containing turmeric, as compared to the polyethylene reference.

Figure 2 shows the results of the FTIR spectroscopic testing for the samples containing turmeric, compared to the polyethylene reference and to the pure turmeric powder. It can be observed that the spectra are dominated by the relatively strong bands of the polymer, with only minor signals appearing due to the presence of turmeric, in regions 2, 4, and 6.

Bands related to polyethylene appear at about 2920 cm${}^{-1}$ (CH_2_ asymmetric stretching), 2850 cm${}^{-1}$ (CH_2_ symmetric stretching), 1475 cm${}^{-1}$ (bending deformation) 1465 cm${}^{-1}$ (bending deformation), 1375 cm${}^{-1}$ (CH_3_ symmetric deformation), 1365 cm${}^{-1}$ (wagging deformation), 1350 cm${}^{-1}$ (wagging deformation), and 730 cm${}^{-1}$ (rocking deformation) [27].

The turmeric spectrum is similar to the ones previously reported in the literature for pure curcumin [28], featuring a relatively strong band at about 3510 cm${}^{-1}$ (phenolic O-H stretching vibration) followed by other intense bands at about 1630 cm${}^{-1}$ (aromatic moiety C=C stretching), 1600 cm${}^{-1}$ (benzene ring stretching vibrations), 1510 cm${}^{-1}$ (C=O and C=C vibrations), 1430 cm${}^{-1}$ (olefinic C-H bending vibrations), 1280 cm${}^{-1}$ (aromatic C-O stretching vibrations), and 1025 cm${}^{-1}$ (C-O-C stretching vibrations). An additional broad band at about 3400 cm${}^{-1}$ is caused by the presence of water molecules.

The spectrum of polyethylene is not much influenced by the presence of the turmeric powders as various intense bands, such as the ones located at about 1630 cm${}^{-1}$ and 1600 cm${}^{-1}$ and related to the benzene ring vibrations, cannot be detected even when the content of turmeric is 5.0%. On the other hand, bands at about 1000 cm${}^{-1}$ and related to the C-O-C stretching increase with the amount of turmeric, as well as bands in the region 2300 cm${}^{-1}$ and associated with the asymmetric stretching of CO_2_.

CO_2_ can be either absorbed by the composite when exposed to air or due to the interaction between curcumin and polyethylene.

**Figure 3:**
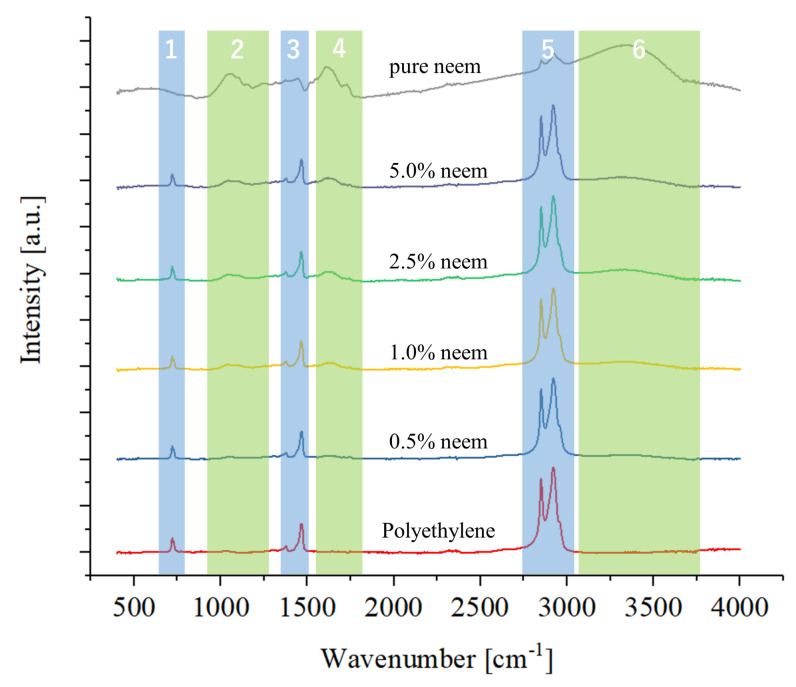
FTIR spectra of the samples containing neem, as compared to the polyethylene reference.

Figure 3 shows the results of the FTIR spectroscopy analysis on the samples containing neem, compared to the same polyethylene reference and to pure neem powder. Unlike curcumin, the presence of neem in the composites can be determined by observing the presence of bands in regions 2, 4, and 6, all of which increase with the concentration.

The spectrum of neem powder is quite similar to the reference for turmeric of Figure 2, but unlike turmeric, neem is composed of many different substances that can contribute to the FTIR spectra, such as: chlorophyll, cellulose, quercetin, isomeldenin, nimbin, nimbinene, 6-desacetyl nimbinene, nimbandiol, immobile, nimocinol, and beta-sitosterol.

Due to the complexity of the system, it is impossible to deconvolve the spectrum into its basic constituents. Nevertheless, it can be observed that the three regions, related to aromatic C-O stretching vibrations (at about 1280 cm${}^{-1}$) and C-O-C stretching (at about 1025 cm${}^{-1}$), to aromatic moiety C=C stretching (at about 1630 cm${}^{-1}$ and 1600 cm${}^{-1}$) and to water gradually, increase with the fraction of neem powder.

**Figure 4:**
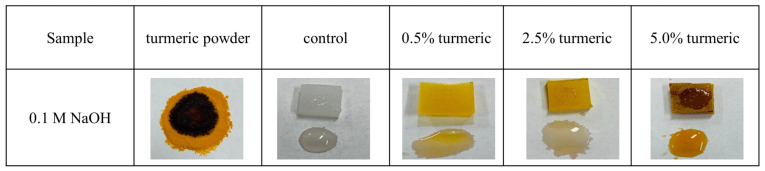
Results of a simple test performed dropping 10 μm of 0.1 M NaOH solution on the surface of the samples, as a function of the concentration of turmeric.

Figure 4 shows the results of a simple test performed by dropping 10 μm of 0.1 M NaOH solution on the surface of the samples. The alkaline solution induces the transformation of curcumin from the diketone to the keto-enol form, changing its color to red. This experiment was used to verify whether the reinforcing powder when exposed to the environment can leech from the composite when in solution.

It can be observed that, even when at low concentrations (0.5 wt.%), the curcumin mixed into the polyethylene matrix is still able to react to the alkaline solution. Moreover, curcumin is released from the composite polymer into the solution, which also becomes red.

For both the composite and solution, the intensity of the color is a function of the fraction of curcumin.

**Figure 5:**
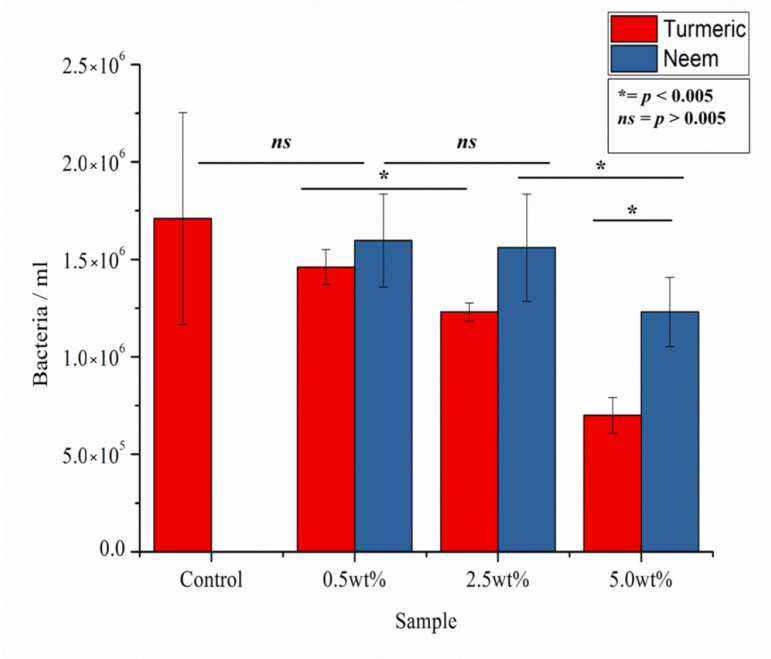
CFU counting as performed on the various samples after testing in vitro with *E. coli* for 24 h.

Figure 5 shows the CFU counting results for the composites tested against *E. coli* for 24 h, as compared to the control culture. It can be observed that the antibacterial effect of turmeric is stronger than neem, even at relatively low concentrations. For neem, only a concentration of about 5.0% wt. was able to show a statistically significant decrease in CFU.

**Figure 6:**
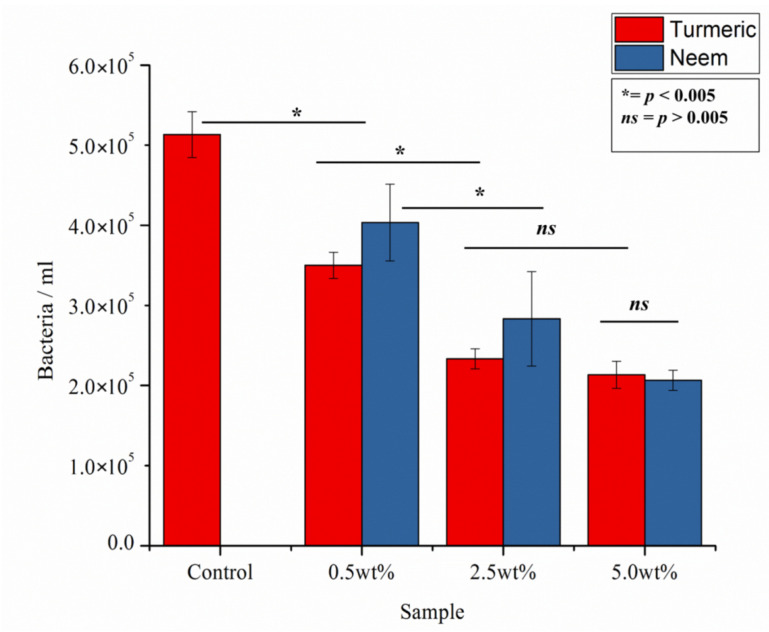
CFU counting as performed on the various samples after testing in vitro with *S. aureus* for 24 h.

Figure 6 shows the CFU counting results for the composites tested against *S. aureus*. Both neem and turmeric show comparable antibacterial properties against this specific strain, and the effects are already statistically significant at low concentrations. Overall, turmeric seems to be more effective than neem, particularly at lower concentrations.

**Figure 7:**
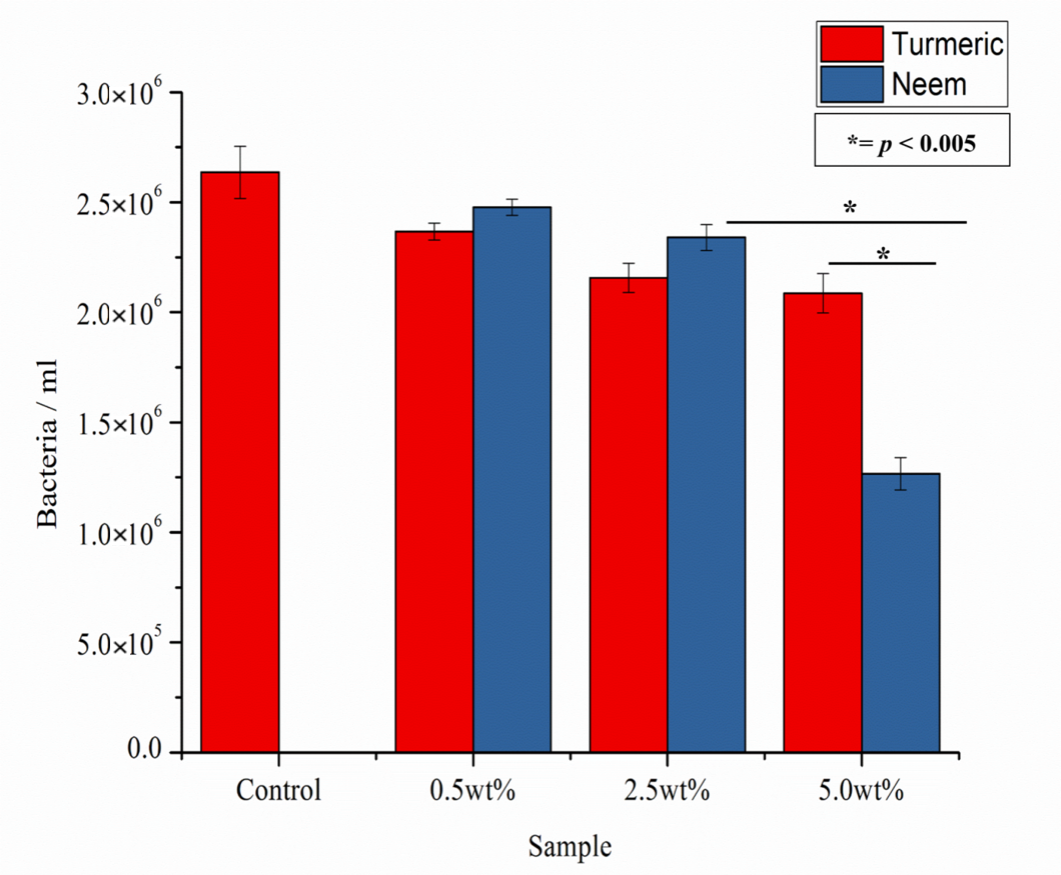
CFU counting as performed on the various samples after testing in vitro with *C. albicans* for 24 h.

Figure 7 shows the CFU counting results for the composites tested against *C. albicans*. Unlike previous results against bacteria, low concentrations of neem and turmeric do not seem to have a statistically relevant anti-fungal effect, and only neem seems to have a strong effect against *C. albicans* at high concentrations.

**Figure 8:**
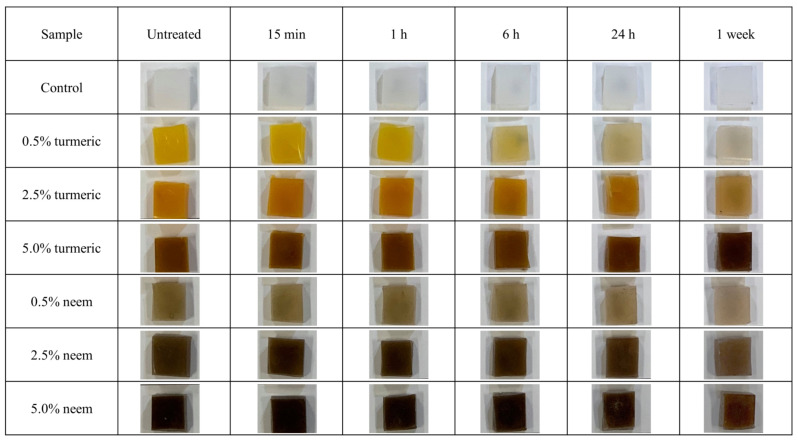
Discoloration of the different samples after UV treatment, as a function of the concentration of bioactive ingredients and the time of UV exposure.

Figure 8 shows the images of the different samples after UV irradiation. It can be observed that the color of both neem and turmeric composites tends to fade away with exposure, in particular for lower contents. For turmeric, this effect happens in two stages, the first being the crosslinking of curcumin with polyethylene, as previously described in the literature [29], followed by a degradation of the curcumin molecule to produce bicyclopentadione, vanillin, ferulic acid, and feruloylmethane [30,31].

During this degradation, the breaking of the C-C bonds in the central chain is responsible for the loss in color emission.

For neem, the original green pigmentation caused by the chlorophyll *a* contained in the leaves is also photochemically degraded into hydroxy-pheophytin *a*, pheophytin *a*, and hydroxy-lactone-pheophytin *a* [32], thus leading to discoloration.

**Figure 9:**
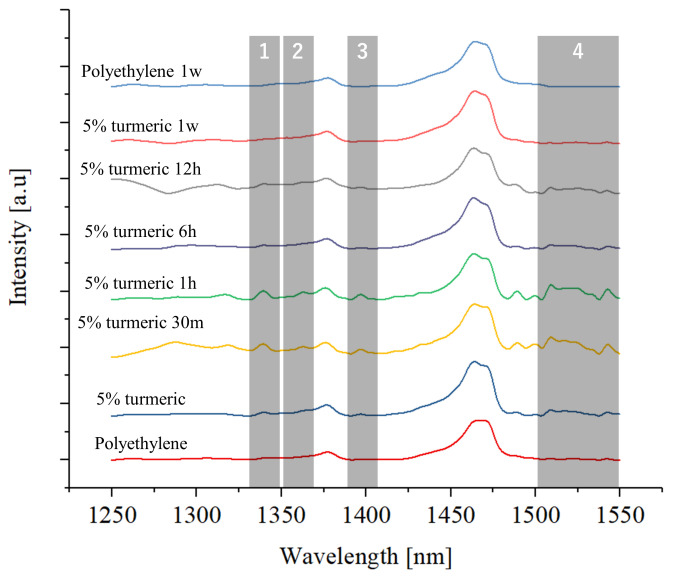
FTIR results for the turmeric sample after exposure to UV, as a function of the time of treatment, as compared with pure polyethylene references.

Figure 9 shows the results of the FITR testing performed after UV aging on the samples containing 5% of turmeric powder. Even if the general shape of the spectra does not differ much from the ones presented in Figure 2, the region between 1250 and 1550 cm${}^{-1}$ presents some minor changes. It can be observed that the bands at about 1340, 1360, and 1390 cm${}^{-1}$ and between 1500 and 1550 cm${}^{-1}$ initially increase in relative intensity after 30 min and 1 h of UV treatment, then decrease and finally disappear after one week. This is caused by the degradation of curcumin into ferulic acid, which has relatively more intense bands in these regions [33]. Ferulic acid is then degraded into smaller sub-products.

**Figure 10:**
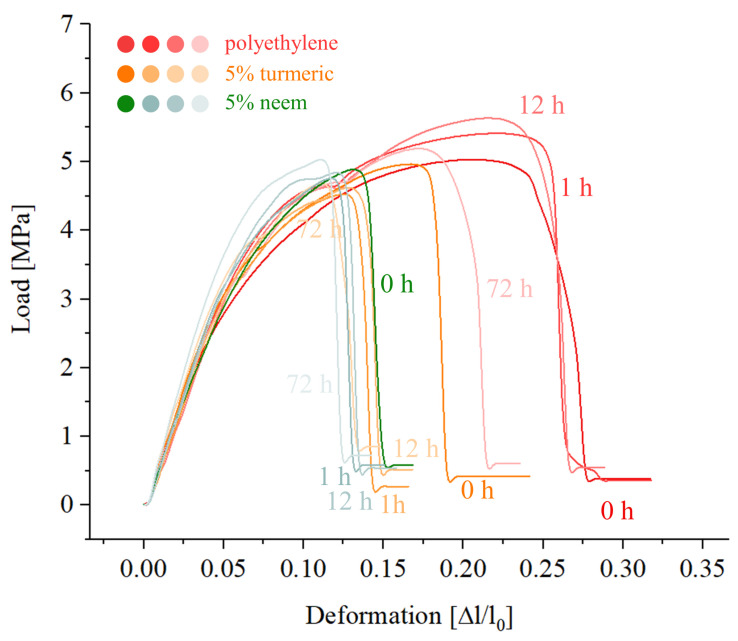
Mechanical properties (tensile) for the samples containing 5% of reinforcing particles, as a function of UV treatment, compared to the polyethylene references.

Mechanical testing results, performed in a tensile configuration on the samples with the highest fractions of reinforcing powders, are presented in Figure 10. It can be observed that UV treatment has initially no effect on polyethylene, but it reduces the maximum deformation from about 27% to 22% after three days of continuous treatment.

The addition of turmeric not only reduces both the maximum load (from 5.5 to 4.7 MPa) and the deformation (from 27% to 18%), but it also makes the composite much more sensible to UV exposure. After just one hour of treatment, the load further decreases to about 4.5 MPa, and the elongation to about 15%; however, the properties stabilize afterwards, and no further decrease is observed for longer exposure times.

Neem seems to have the greatest influence on the mechanical properties, reducing the elongation from 27% to about 15%, but the values are not much affected by UV exposure.

## 4. Discussion

The bioactive molecule responsible for most of the biological effects in turmeric as reported in the literature is curcumin. It has been postulated that the small curcumin molecule is able to penetrate both Gram positive and Gram negative bacteria, leading to a permanent damage of the membrane, which causes leakage [34]. Moreover, it was shown that curcumin can suppress the bacterial cell proliferation by the inhibition of the assembly dynamics of FtsZ protein (encoded by the *ffsZ* gene) in the Z-ring [35].

Even if the two bacteria strains, *E. coli* (Gram negative) and *S. aureus* (Gram positive), have different membrane structures, curcumin was able to penetrate both, as suggested by the simple test of Figure S2. Five milliliters of PBS were used to wash the samples, and a small amount (100 μL) was used for the CFU counting. In the case of bacterial solutions, the rest of the volume was collected in three different microcentrifuge tubes (5 mL each). Centrifugation was conducted twice using the high-speed refrigerated centrifuge, CR-GIII (Hitachi Ltd, Tokyo, Japan), at 4 ${}^{{}^{\circ}}$C and 10,000 rpm for 1 min in order to remove the turmeric or neem powders released by the surface. After centrifugation and repeated washing, the bacteria pellets still contained particles of turmeric, resulting in a yellow color that could not be washed off and did not turn red when exposed to solutions containing NaOH. This indicates how bacteria could have absorbed the turmeric from the samples.

For neem-reinforced composites, the antibacterial effect was supposed to be the result of the presence of both quercetin and sitosterol [36]. While neem extracts were successfully used as antibacterial agents against Gram positive strains [37], previous literature results have shown that they are not as effective against Gram negative strains [38], as confirmed by comparing the results of Figure 5 and Figure 6.

On the other hand, neem has shown greater efficiency against fungal infection of *C. albicans*. The antifungal effect of neem powder is also associated with the presence of quercetin, which has shown effects against various strains of Candida [39].

The drawback of using neem or turmeric powders to produce a polymer matrix composite is the decrease of the mechanical properties. Both turmeric and neem cause a decrease of the mechanical properties, particularly elongation, with a loss of strength and elongation comparable to other polymer matrix composites from the literature [40].

The better performances of turmeric-reinforced polyethylene when compared to neem-reinforced can be explained by considering the capability of curcumin to bond to the chains of polyethylene, resulting in a higher interface adhesion between the particles and the matrix.

Combining polyethylene with turmeric makes the composite material more sensitive to UV irradiation, leading to a reduction of the mechanical properties over time. Even if the loss of the mechanical properties was somehow limited, short time UV irradiation did not improve the mechanical performances as in the case of pure polyethylene.

Mechanical testing proved that the structure of turmeric-polyethylene composites can be degraded with UV exposure, but the degradation was not strong enough to lead to decomposition.

## 5. Conclusions

In this research, up to 5% of natural products, namely turmeric and neem powders, were successfully added to polyethylene in order to obtain a composite material.

Both neem and turmeric powders improve the antibacterial efficiency of polyethylene, with turmeric showing efficiency against both Gram positive and negative strains.

Neem-based composites, on the other hand, were more effective against *C. albicans* fungal colonization.

The mechanical strength of polyethylene was compromised by the presence of reinforcing particles, decreasing the maximum load and, more specifically, the elongation.

## Figures and Tables

**Table 1:** Samples’ composition.

Turmeric	Neem
wt.%	wt.%
0% (reference)	
0.5%	0.5%
1.0%	1.0%
2.5%	2.5%
5.0%	5.0%

## Supplementary Materials

The following are available online at https://www.mdpi.com/2079-6382/9/12/857/s1.
