# Supplementary material for: Antibacterial and Antifungal Properties of Composite Polyethylene Materials Reinforced with Neem and Turmeric
Source: Antibiotics (Basel). 2020 Nov 30;9(12):857. doi: 10.3390/antibiotics9120857 (PMC7760416; doi:10.3390/antibiotics9120857)
Supplement: Supplementary file 1 [file antibiotics-09-00857-s001.pdf]

antibiotics

Article

Antibacterial and Antifungal Properties of Composite Polyethylene Materials Reinforced with Neem and Turmeric

Thefye P. M. Sunthar [1,2], Elia Marin [1,3,*], Francesco Boschetto [1,2,3], Matteo Zanocco [1,2], Hirofumi Sunahara [4], Raviduth Ramful [5,6], Kaeko Kamei [4], Wenliang Zhu [1] and Giuseppe Pezzotti [1,2,7,8]

1 Ceramic Physics Laboratory, Kyoto Institute of Technology, Sakyo-ku, Matsugasaki, Kyoto 606-8585, Japan; d0871502@edu.kit.ac.jp (T.P.M.S.); boschetto-cesc@kit.ac.jp (F.B.); d8871004@edu.kit.ac.jp (M.Z.); wlzhu@kit.ac.jp (W.Z.); pezzotti@kit.ac.jp (G.P.)
2 Department of Immunology, Graduate School of Medical Science, Kyoto Prefectural University of Medicine Kamigyo-ku, 465 Kajii-cho, Kawaramachi dori, Kyoto 602-0841, Japan
3 Department of Dental Medicine, Graduate School of Medical Science, Kyoto Prefectural University of Medicine, Kamigyo-ku, Kyoto 602-8566, Japan
4 Department of Biomolecular Engineering, Kyoto Institute of Technology, Sakyo-ku, Matsugasaki, Kyoto 606-8585, Japan; m9674018@edu.kit.ac.jp (H.S.); kame@kit.ac.jp (K.K.)
5 Graduate School of Science and Technology, Kyoto Institute of Technology (KIT), Matsugasaki, Sakyo-ku, Kyoto 606-8585, Japan; r.ramful@uom.ac.mu
6 Mechanical and Production Engineering Department, Faculty of Engineering, University of Mauritius, Reduit 80837, Mauritius;
7 The Center for Advanced Medical Engineering and Informatics, Osaka University, Yamadaoka, Suita, Osaka 565-0871, Japan
8 Department of Orthopedic Surgery, Tokyo Medical University, Tokyo, 105-8461, Japan
* Correspondence: elia-marin@kit.ac.jp

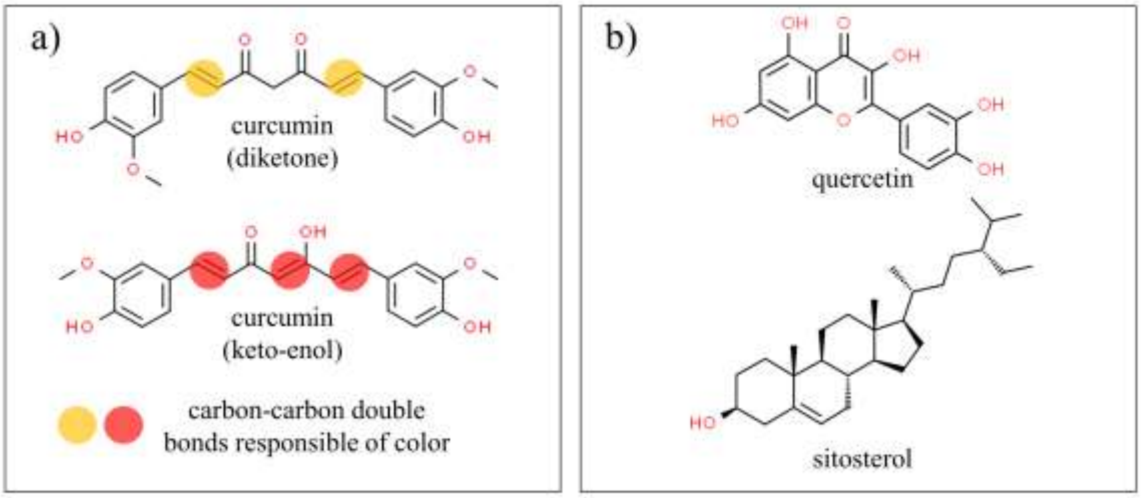

Figure S1. Molecular structure of the bioactive compounds of (**a**) turmeric, in both diketone and keto-enol form and (**b**) neem, quercetin and sitosterol.

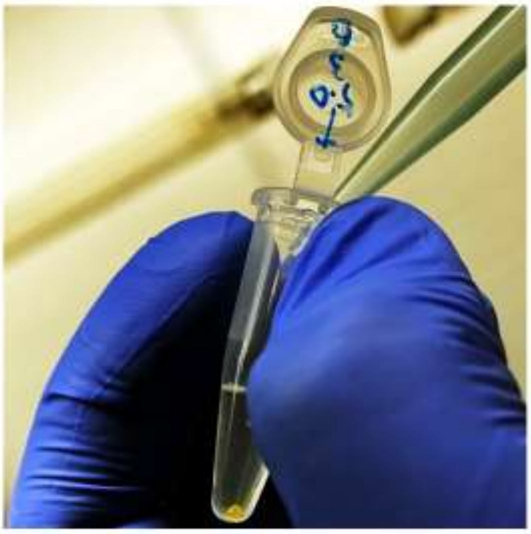

Figure S2. Centrifuged pellet of E. coli bacteria exposed to 5% turmeric polyethylene composite showing the presence of curcumin inside the cell membranes.
